# Socioeconomic Position and DNA Methylation Age Acceleration Across the Life Course

**DOI:** 10.1093/aje/kwy155

**Published:** 2018-07-27

**Authors:** Amanda Hughes, Melissa Smart, Tyler Gorrie-Stone, Eilis Hannon, Jonathan Mill, Yanchun Bao, Joe Burrage, Leo Schalkwyk, Meena Kumari

**Affiliations:** 1 Institute for Social and Economic Research, University of Essex, Colchester, United Kingdom; 2 Department of Biological Sciences, Faculty of Science and Health, University of Essex, Colchester, United Kingdom

**Keywords:** aging, DNA methylation, epigenomics, socioeconomic factors

## Abstract

Accelerated DNA methylation age is linked to all-cause mortality and environmental factors, but studies of associations with socioeconomic position are limited. Researchers generally use small selected samples, and it is unclear how findings obtained with 2 commonly used methods for calculating methylation age (the Horvath method and the Hannum method) translate to general population samples including younger and older adults. Among 1,099 United Kingdom adults aged 28–98 years in 2011–2012, we assessed the relationship of Horvath and Hannum DNA methylation age acceleration with a range of social position measures: current income and employment, education, income and unemployment across a 12-year period, and childhood social class. Accounting for confounders, participants who had been less advantaged in childhood were epigenetically “older” as adults: In comparison with participants who had professional/managerial parents, Hannum age was 1.07 years higher (95% confidence interval: 0.20, 1.94) for participants with parents in semiskilled/unskilled occupations and 1.85 years higher (95% confidence interval: 0.67, 3.02) for those without a working parent at age 14 years. No other robust associations were seen. Results accord with research implicating early life circumstances as critical for DNA methylation age in adulthood. Since methylation age acceleration as measured by the Horvath and Hannum estimators appears strongly linked to chronological age, researchers examining associations with the social environment must take steps to avoid age-related confounding.

Epigenetics is the study of chemical modifications to DNA and the histone proteins bound to it, which play an important regulatory role in gene expression without changing the heritable DNA sequence. The most widely studied epigenetic modification in relation to human health and disease is DNA methylation. In recent years a number of mathematical models predicting age from DNA methylation profiles, or “epigenetic clocks,” have been developed, including those by Horvath (1) and Hannum et al. (2). Utilizing age-related changes in DNA methylation which occur throughout the life span, these models allow calculation of a person’s DNA methylation age (DNAm age) based on methylation at a small number of selected sites (<0.1% of sites available from microarrays used to profile DNA methylation; <0.001% of 5′-C–phosphate–G-3′ (CpG) sites in the human genome (3)). DNAm can therefore be considered a measure of “biological age” (2).

Although DNAm age and chronological age are highly correlated, the relationship varies between individuals, such that some people are “older” in DNA methylation terms than their chronological age would predict and others are “younger.” This variation is often described in terms of “delta age” (Δage), the difference in years between a person’s DNAm age and his or her chronological age. People with unexpectedly high DNAm age are said to show DNA methylation “age acceleration” (although, since Δage characterizes a difference rather than a rate of change, a more appropriate term might be “elevation”). Interpersonal variation in Δage is associated with functioning among elderly people—walking speed, lung function, and cognition (4)—and all-cause mortality (5, 6). Persons with higher Horvath or Hannum DNAm age are at increased risk of age-related mortality, and persons with younger methylation profiles are at lower risk. Strikingly, this applies even within pairs of twins (7). Meanwhile, DNAm age acceleration has been linked with environmental factors, including economic hardship (8), lifetime stress (9), dietary factors (10), pollution (11), and education (12). This suggests that DNAm age acceleration may reflect processes contributing to social differences in morbidity and mortality, opening up new pathways of inquiry in health inequalities research. A mediating role of stress (13) is plausible given existing research on socioeconomic disadvantage, stress, and adverse aging profiles (14).

Another area of ongoing research concerns the applicability of the clocks to population samples with different age ranges than those on which the clocks were calibrated. The Horvath clock, based on 8,000 samples encompassing different tissues taken from participants aged ≤100 years of different ethnicities (1) and the Hannum clock, calculated from blood samples of 666 white or Hispanic-American adults (2), obtain linear relationships between chronological age and DNAm age in their samples. However, it is unclear how well this relationship holds in populations with different age distributions. A recent analysis by Zhang et al. (15), using a German sample aged 50–75 years, suggested that DNAm age calculated using the clocks may predict chronological age less well at older ages. If the relationship between chronological age and DNAm age is not constant during adulthood, this must be considered in analysis of possible “accelerators” which are age-patterned, to avoid age-related confounding.

To investigate the possible contribution of DNAm age acceleration to socioeconomic inequalities in health, we examined the relationship of DNAm age with a range of socioeconomic measures in 1,099 United Kingdom men and women aged 28–98 years. We investigated Horvath age and Hannum age, the most widely used measures of DNAm age, in parallel. Since socioeconomic position has multiple dimensions whose associations with health may differ (16) and previous epigenetic studies suggest that accumulation processes may exert particular effects (9), we considered contemporaneous factors (current employment status and income), cumulative measures (income and total unemployment over 12 years), and factors from earlier in life (educational qualifications and parental social class at age 14 years). We also investigated whether the relationship between DNAm age and chronological age changed across the adult life span.

## METHODS

### Participants

The British Household Panel Survey began in 1991, and in 2010 it was incorporated into a larger study, Understanding Society: The UK Household Longitudinal Study (17). Since 1991, sociodemographic information has been collected through annual interviews, and in 2011–2012, blood samples for British Household Panel Survey participants were collected during a nurse visit in the participant’s home. Respondents were eligible to give a blood sample if they were aged 16 years or more, were not pregnant, and met other conditions detailed in the UK Household Longitudinal Study user’s guide (18). Funding was not available to profile DNA methylation in all participants who gave necessary consent. Therefore, methylation was profiled in DNA extracted from whole blood for 1,193 persons who were eligible for and consented to blood sampling and genetic analysis, who participated in all annual interviews between 1999 and 2011, and whose time between blood sample collection and processing did not exceed 3 days. Eligibility requirements for genetic analyses meant that the epigenetic sample was restricted to participants of white ethnicity. Eighteen persons were excluded following laboratory quality control checks. The current analysis excluded 76 participants for whom inverse-probability weights could not be calculated and outliers whose Δage exceeded 3 standard deviations from the mean (*n* = 6 for Horvath analyses, *n* = 5 for Hannum analyses). Pairwise deletion for missing data resulted in a minimum sample size of 932 for a summary measure of net household income over the 12-year period.

### Measures

#### DNA methylation

Five hundred–nanogram samples of whole-blood DNA from 1,193 persons were treated with sodium bisulfite using the EZ96 DNA methylation kit (Zymo Research, Irvine, California) following the manufacturer’s standard protocol. DNA methylation was assessed using the Illumina Infinium HumanMethylationEPIC BeadChip kit (Illumina, Inc., San Diego, California) (19). DNA methylation levels were quantified on an Illumina HiScan System (Illumina, Inc.). Raw signal intensities were parsed into R (R Foundation for Statistical Computing, Vienna, Austria) and converted into β values using the Bioconductor bigmelon package (20).

Outliers were identified and removed using “wateRmelon::outlyx,” low-quality samples (<85% bisulfite conversion) were identified and removed using “wateRmelon::bscon,” and data were normalized using “wateRmelon::dasen.” Differences between normalized and raw data were estimated using “wateRmelon::qual.” Observations with a root mean square difference and standard deviation of difference greater than 0.05 were removed. After removal of outlying/poor-quality observations, data were subjected to “wateRmelon::pfilter” and renormalized using “wateRmelon::dasen,” leaving 857,071 probes and 1,175 persons for analysis.

DNAm age was calculated through linear functions using wateRmelon::agep, supplying different sets of coefficients for Horvath or Hannum calculations following

$$\begin{array}{l} {\mathrm{Age}}_{\mathrm{sample}}=m_{\mathrm{probe}\;1}\beta_{\mathrm{probe}\;1}+m_{\mathrm{probe}\;2}\beta_{\mathrm{probe}\;2}\\ \;+\;\cdots \;+\;m_{\mathrm{probe}\;n}\beta_{\mathrm{probe}\;n}+c, \end{array}$$

where *m* is the coefficient of the specified probe, β is the measurement of DNA methylation for a specified probe and a given individual, and *c* is the intercept defined by the author’s model. Because both clocks were designed for an earlier microarray design, missing probes (17 for Horvath, 6 for Hannum; listed in Web Table 1, available at https://academic.oup.com/aje) were not included in calculations.

Horvath and Hannum Δage were calculated as the difference between DNAm and chronological age and were included in linear regression models as the dependent variable.

#### Socioeconomic measures

Measures of current socioeconomic position were based on participants’ self-reported data from the annual interview preceding the 2011 nurse visit, while lifetime measures used information given during the previous 12 years. For current income, quartiles of equivalized net household income in 2011 were calculated separately within 5-year age groups, given substantial age-group differences in household income (2-sided *P* < 0.01), which increased to age 60 years before decreasing sharply around retirement age. Equivalently, a summary income measure for the 12-year period considered years spent in the lowest age-group–specific quartile (categorized as 0, 1–2, 3–6, or ≥7 for roughly equal groups). Current employment status was categorized as employed, self-employed, unemployed, retired, looking after home or family, long-term sick or disabled, or other; unlike the case for other measures, this analysis was restricted to participants of working age (≤65 years). Reports of current and former employment and nonemployment spells from each annual wave were used to construct 12-year activity histories for each participant, from which aggregated duration of unemployment (in months) was calculated; this was categorized as none, ≤12 months, or >12 months.

Highest educational qualification was categorized as university degree, qualifications below university degree, or no qualifications. Following the method of Fiorito et al. (12), the variable was standardized within each sex and 5-year age group to account for generational differences in education; this resulted in a continuous score between 0 and 1, with higher scores indicating less education relative to other participants within the same age and sex group. Childhood social class was based on parents’ Registrar General’s Social Classification when participants were 14 years of age and was categorized as professional/managerial occupations, skilled nonmanual occupations, skilled manual occupations, or semiskilled/unskilled occupations. The social class of the father was used unless this information was not available, in which case the mother’s social class was used instead. In cases where neither parent was employed or both parents were deceased, participants were assigned to a separate group.

#### Covariates

All analyses included as covariates sex, chronological age, age^2^ (to capture possible nonlinearity in age-related confounding), smoking, and adiposity. All analyses adjusted for batch and white blood cell composition estimates, which were calculated using the Houseman reference-based algorithm implemented in the estimateCellCounts function package in minfi (21, 22).

Smoking was categorized as never smoker, ex-smoker, current smoker of ≤10 cigarettes/day, current smoker of 11–20 cigarettes/day, or current smoker of ≥21 cigarettes/day. Adiposity was indexed using World Health Organization classifications of body mass index (weight (kg)/height (m)^2^): underweight (<18.5), normal-weight (18.5–24.9), overweight (25.0–29.9), class I obese (30.0–34.9), or class II obese (≥35.0) (23). Because of substantial data missingness, alcohol consumption in the past week and psychological distress were examined in sensitivity analyses only. These analyses considered past-week alcohol drinking frequency (most days, 3–4 days, 1–2 days, or none) and intensity (none, under the recommended limit, at or above the recommended limit, under twice the recommended limit, or at least 2 times the recommended limit). Psychological distress was assessed at the interview preceding the nurse visit using the 12-item General Health Questionnaire (24), scored continuously from 0 to 36.

Analyses were conducted in STATA, version 15 (StataCorp LP, College Station, Texas). Analyses used inverse-probability weights to account for both unequal initial sampling probabilities and differential attrition and nonresponse (Web Appendix), accounting for survey design effects using the *svyset* command.

## RESULTS

### Description of the sample

The analytical sample included for DNA methylation analysis (Table 1) was indirectly selected on the basis of age, since DNA methylation was profiled only for persons who had participated as adult (age ≥16 years) survey respondents annually since 1999. Compared with white/European British Household Panel Survey participants who took part in the nurse assessment and were in the eligible age range (age ≥28 years) but were not in the analytical sample, participants included in analyses did not differ significantly (at *P* < 0.05) with regard to sex, mean body mass index, aggregated unemployment, or childhood social class. They were, however, older (58.4 years vs. 53.9 years), less educated (28.7% with a university degree vs. 30.8%; 17.6% with no qualifications vs. 14.4%) and had a lower equivalized net household income (£1,599.2/month (approximately $2,558.72/month in March 2012) vs. £1,978.6/month (approximately $3,165.76/month in March 2012) (25)). They were less likely to be employed (41.0% vs. 50.8%), more likely to be retired (40.8 vs. 29.4%), and less likely to be current smokers (15.7% vs. 20.2%) (all *P* values < 0.05). Childhood social class predicted adult socioeconomic position (Web Table 2).

**Table 1.** Characteristics of the Analytical Sample in a Study of DNA Methylation Age (*n* = 1,099), UK Household Longitudinal Study, Wave 3, 2011–2012

Characteristic	No. of Persons	%
Age, years^a,b^	58.4 (14.9)	
Sex		
Male	466	42.4
Female	633	57.6
Current employment status		
Employed	450	41.0
Self-employed	93	8.5
Unemployed	23	2.1
Retired	448	40.8
Looking after home or family	47	4.3
Long-term sick or disabled	31	2.8
Other	7	0.6
Highest educational qualification		
University degree	315	28.7
Qualifications below degree	586	53.3
No qualifications	193	17.6
Missing data	5	0.5
Total duration of unemployment (1999–2011), months		
0	923	84.0
<12	114	10.4
≥12	60	5.5
Missing data	2	0.2
Childhood social class^c^		
Professional/managerial	281	25.6
Skilled nonmanual	104	9.5
Skilled manual	406	36.9
Semiskilled/unskilled	192	17.5
No employed parent/both parents deceased	48	4.4
Missing data	68	6.2
Smoking		
Never smoker	594	54.1
Ex-smoker	332	30.2
Current smoker, cigarettes/day		
≤10	60	5.5
11–20	90	8.2
≥21	23	2.1
Body mass index^d^ category		
Underweight (<18.5)	6	0.6
Normal-weight (18.5–24.9)	303	27.6
Overweight (25.0–29.9)	449	40.9
Obese class I (30.0–34.9)	227	20.7
Obese class II (≥35)	114	10.4

Abbreviation: UK, United Kingdom.

^a^ Values are presented as mean (standard deviation).

^b^ Age range, 28–98 years.

^c^ Parental Registrar General’s Social Classification when participant was aged 14 years.

^d^ Weight (kg)/height (m)^2^.

### Correlation of chronological age and DNAm age across the adult life span

**Figure 1.**
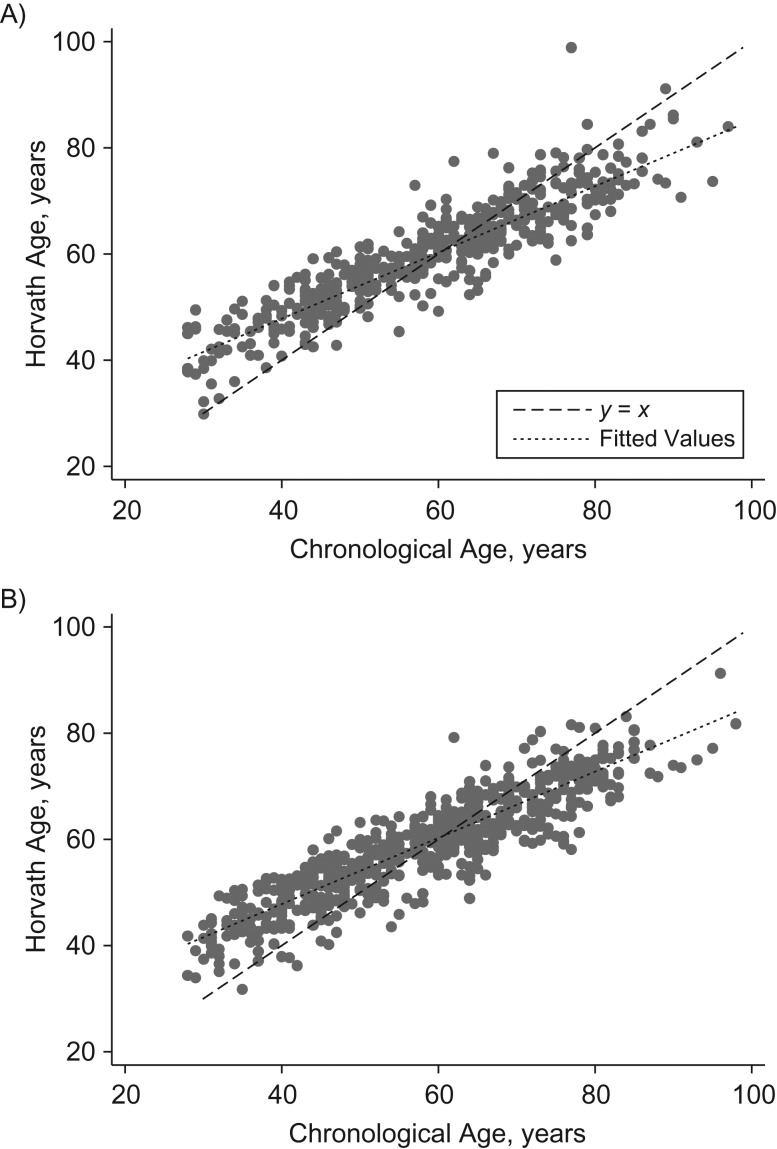
Horvath DNA methylation age (years) according to chronological age (years) among men (A) and women (B) in the UK Household Longitudinal Study (*n* = 1,093), 2011–2012. The solid line represents the line of best fit, and the dashed line is the *y* = *x* line. ΔAge (years) is the difference between DNA methylation age and chronological age (i.e., the vertical distance from a dot to the *y* = *x* line). If mean Δage were constant with age, observations would be approximately symmetrical about the *y* = *x* line. Instead, Δage decreases with chronological age. UK, United Kingdom.

**Figure 2.**
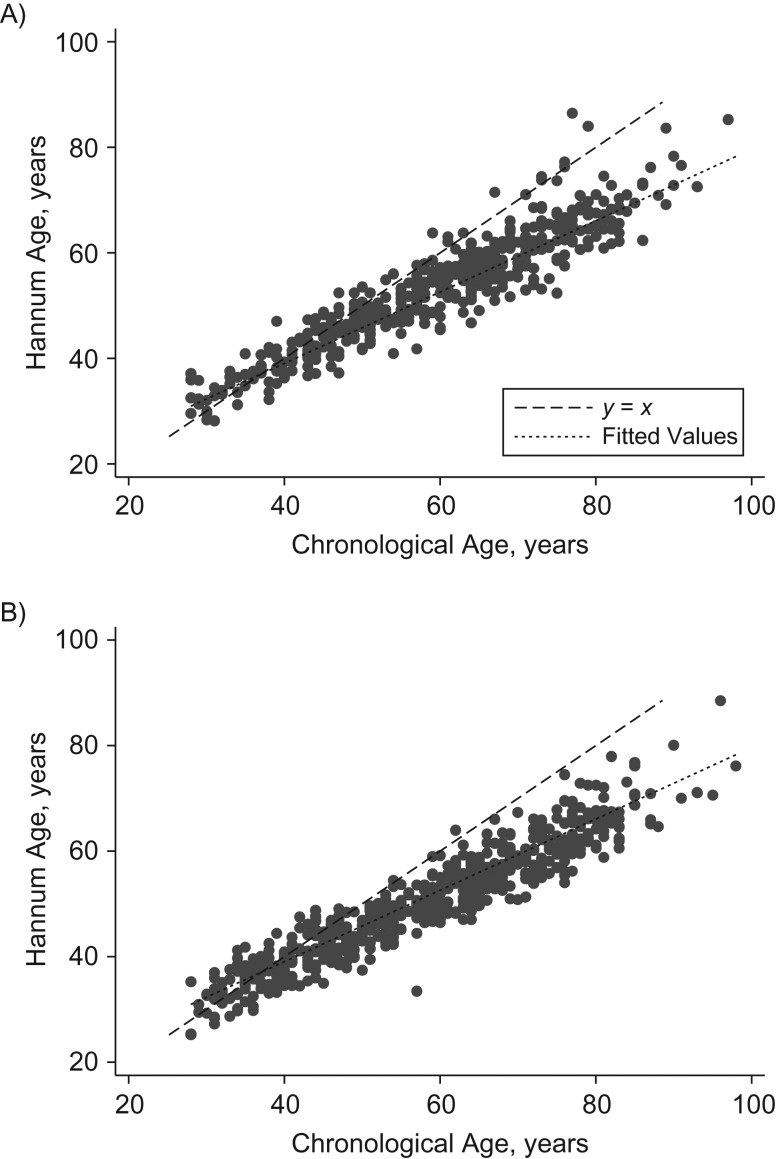
Hannum DNA methylation age (years) according to chronological age (years) among men (A) and women (B) in the UK Household Longitudinal Study (*n* = 1,094), 2011–2012. The solid line represents the line of best fit, and the dashed line is the *y* = *x* line. ΔAge (years) is the difference between DNA methylation age and chronological age (i.e., the vertical distance from a dot to the *y* = *x* line). If mean Δage were constant with age, observations would be approximately symmetrical about the *y* = *x* line. Instead, Δage decreases with chronological age. UK, United Kingdom.

Across the sample, chronological age correlated highly with Horvath DNAm age (*r* = 0.90) and Hannum DNAm age (*r* = 0.94). Horvath and Hannum DNAm age were also highly correlated (*r* = 0.92). However, scatterplots of DNA methylation with chronological age (Figures 1 and 2) showed that the relationship between DNAm age and chronological age differed substantially by chronological age. Using the Horvath clock, the youngest participants were substantially older in terms of DNA methylation than chronologically, whereas for the older participants the reverse was true. Using the Hannum clock, DNA methylation and chronological age correlated well for younger participants, but older participants were substantially younger in DNA methylation terms than chronologically. In weighted regression models adjusting for sex, batch, and white blood cell composition, regression coefficients for Δage against chronological age were negative and significant for both clocks, and they did not differ by sex (using the Horvath method, coefficients were −0.39 (95% confidence interval (CI): 0.42, 0.36) for all participants, −0.39 (95% CI: 0.41, 0.36) for men, and −0.39 (95% CI: 0.42, 0.37) for women; using the Hannum method, coefficients were −0.38 (95% CI: 0.40, 0.36) for all participants, −0.37 (95% CI: 0.39, 0.35) for men, and −0.38 (95% CI: 0.40, 0.36) for women). The addition of quadratic age terms did not indicate substantial nonlinearity (for the Horvath and Hannum methods, age^2^ coefficients were −0.0007 (2-sided *P* = 0.39) and −0.0003 (2-sided *P* = 0.67), respectively).

### Association of DNAm age acceleration with socioeconomic factors

#### Horvath DNAm age

Using the Horvath clock (Table 2), in adjusted models no significant associations were seen between Δage and current income, income over the 12-year period, educational qualifications, or aggregated unemployment. Participants not working due to sickness or disability were 1.89 years “older” (95% CI: 0.40, 3.37) than their employed counterparts; no other employment status differences were observed. Only childhood social class showed clear elevations for less advantaged groups (Table 2). Compared with participants with professional/managerial parents, Δage for participants with parents in skilled nonmanual occupations was 1.42 years higher (95% CI: 0.24, 2.59), and for participants with no employed parent or both parents deceased at age 14 years, Δage was 2.40 years higher (95% CI: 0.60, 4.19). Treating childhood social class as continuous showed a significant increasing Δage across groups (per-category Δage change = 0.33 year, 95% CI: 0.06, 0.59). The addition of alcohol measures or psychological distress to the model reduced sample sizes and hence precision but did not affect conclusions (Table 3).

**Table 2.** Association^a^ of Socioeconomic Factors With DNA Methylation Age Acceleration Among Participants (*n* = 1,094) in the UK Household Longitudinal Study, 2011–2012

Socioeconomic Factor	Horvath Method		No. of Persons in Model	Hannum Method		No. of Persons in Model
	ΔAge, years	95% CI		ΔAge, years	95% CI	
Quartile of equivalized net household income						
4 (highest)	0	Referent	1,093	0	Referent	1,094
3	−0.88	−1.84, 0.07		−0.36	−1.04, 0.31	
2	0.44	−0.49, 1.37		0.12	−0.62, 0.86	
1 (lowest)	−0.68	−1.61, 0.25		−0.17	−1.00, 0.65	
Current employment status (participants aged ≤65 years)						
Employed	0	Referent	716	0	Referent	717
Self-employed	0.81	−0.39, 2.01		0.07	−0.93, 1.06	
Unemployed	−0.97	−2.99, 1.05		−0.88	−2.14, 0.37	
Retired	−0.65	−1.86, 0.57		−0.33	−1.33, 0.66	
Looking after home or family	0.98	−0.35, 2.31		0.52	−0.53, 1.57	
Long-term sick or disabled	1.89	0.40, 3.37		−0.37	−1.83, 1.08	
Other	1.35	−0.78, 3.48		0.92	−1.69, 3.52	
Duration of time in the lowest age-specific income quartile (1999–2011), years						
0	0	Referent	932	0	Referent	933
1–2	0.34	−0.55, 1.23		0.68	−0.13, 1.48	
3–6	−0.46	−1.28, 0.36		−0.01	−0.64, 0.62	
≥7	−0.73	−1.54, 0.08		−0.24	−0.94, 0.46	
Total duration of unemployment (1999–2011), months						
0	0	Referent	1,091	0	Referent	1,092
<12	−0.72	−1.70, 0.26		−0.45	−1.22, 0.32	
≥12	−0.26	−1.76, 1.25		−0.92	−1.85, 0.01	
Highest educational qualification^b^						
Least educated vs. most educated	0.26	−0.97, 1.49	1,088	0.98^c^	0.03, 1.93	1,089
Childhood social class^d^						
Professional/managerial	0	Referent	1,025	0	Referent	1,026
Skilled nonmanual	1.42^c^	0.24, 2.59		0.33	−0.51, 1.17	
Skilled manual	0.44	−0.30, 1.19		0.68^c^	0.11, 1.25	
Semiskilled/unskilled	0.85	−0.08, 1.79		1.07^c^	0.20, 1.94	
No employed parent/both parents deceased	2.40^c^	0.60, 4.19		1.85^c^	0.67, 3.02	

Abbreviations: CI, confidence interval; UK, United Kingdom.

^a^ Adjusted for chronological age, age^2^, sex, white blood cell composition, batch, smoking, and body mass index.

^b^ Standardized within categories of sex and 5-year age group. Range, 0–1; higher scores indicate lower education.

^c^*P* < 0.05.

^d^ Parental Registrar General’s Social Classification when participant was aged 14 years.

**Table 3.** Results of Sensitivity Analyses for Childhood Social Class and Education in a Study of DNA Methylation Age, UK Household Longitudinal Study, 2011–2012

Variable	Sensitivity Analysis							
	Adjustment for Alcohol Drinking Frequency^a^		Adjustment for Alcohol Drinking Intensity^b^		Adjustment for Psychological Distress^c,d^		Mutual Adjustment for Childhood Social Class and Education^e^	
	ΔAge, years	95% CI	ΔAge, years	95% CI	ΔAge, years	95% CI	ΔAge, years	95% CI
DNAm age calculation method and childhood social class^f^								
Horvath method^g^								
Skilled nonmanual	1.35^h^	0.09, 2.60	1.41^h^	0.14, 2.68	1.35^h^	0.14, 2.57	1.42^h^	0.24, 2.59
Skilled manual	0.24	−0.57, 1.04	0.41	−0.39, 1.21	0.49	−0.28, 1.25	0.45	−0.31, 1.20
Semiskilled/unskilled	0.41	−0.62, 1.45	0.58	−0.46, 1.63	1.12^h^	0.17, 2.07	0.84	−0.15, 1.84
No employed parent/both parents deceased	2.47^h^	0.61, 4.33	2.52^h^	0.61, 4.43	2.40^h^	0.62, 4.18	2.42^h^	0.62, 4.21
Hannum method^i^								
Skilled nonmanual	0.36	−0.48, 1.21	0.43	−0.44, 1.30	0.18	−0.67, 1.04	0.30	−0.54, 1.13
Skilled manual	0.75^h^	0.12, 1.39	0.75^h^	0.12, 1.39	0.49	−0.11, 1.09	0.62^h^	0.06, 1.19
Semiskilled/unskilled	0.86	−0.09, 1.81	0.85	−0.11, 1.81	1.11^h^	0.25, 1.97	0.96^h^	0.06, 1.87
No employed parent/both parents deceased	1.86^h^	0.60, 3.13	1.80^h^	0.51, 3.09	1.83^h^	0.63, 3.04	1.77^h^	0.58, 2.96
Highest educational qualification (Hannum method)^j^								
Lowest education vs. highest education	1.08^h^	0.06, 2.10	0 0.97	−0.07, 2.02	0.78	−0.15, 1.70	0.56	−0.48, 1.60

Abbreviations: CI, confidence interval; DNAm age, DNA methylation age; UK, United Kingdom.

^a^ Results were adjusted for past-week alcohol drinking frequency (most days, 3–4 days, 1–2 days, or none), chronological age, age^2^, sex, white blood cell composition, batch, smoking, and body mass index category.

^b^ Results were adjusted for past-week alcohol drinking intensity (none, under the recommended limit, at or above the recommended limit, under twice the recommended limit, or at least 2 times the recommended limit), chronological age, age^2^, sex, white blood cell composition, batch, smoking, and body mass index category.

^c^ Psychological distress was assessed at the interview preceding the nurse visit using the 12-item General Health Questionnaire (24), scored continuously from 0 to 36.

^d^ Results were adjusted for psychological distress, chronological age, age^2^, sex, white blood cell composition, batch, smoking, and body mass index category.

^e^ Results were mutually adjusted for childhood social class and highest educational qualification, plus chronological age, age^2^, sex, white blood cell composition, batch, smoking, and body mass index category.

^f^ Parental Registrar General’s Social Classification when participant was aged 14 years.

^g^ Analytical sample sizes were *n* = 924, *n* = 904, *n* = 964, and *n* = 1,025, respectively.

^h^*P* < 0.05.

^i^ Analytical sample sizes were *n* = 925, *n* = 905, *n* = 965, and *n* = 1,026, respectively.

^j^ Standardized within categories of sex and 5-year age group. Range, 0–1; higher scores indicate lower education. Analytical sample sizes were *n* = 977, *n* = 957, *n* = 1,026, and *n* = 1,025, respectively.

#### Hannum DNAm age

Using the Hannum clock (Table 2), no differences were seen for employment status, current income, income over the 12-year period, or aggregated unemployment. In contrast, there was a significant association between lower education and higher Hannum age (Δage = 0.98 year, 95% CI: 0.03, 1.93) when comparing the least educated persons with the most educated within age and sex groups, and clear stepwise associations were seen with childhood social class. Compared with participants with professional/managerial parents, Δage for participants with parents in skilled manual occupations was 0.68 year higher (95% CI: 0.11, 1.25), Δage for participants with parents in semiskilled/unskilled occupations was 1.07 years higher (95% CI: 0.20, 1.94), and Δage for participants with no employed parent or both parents deceased at age 14 years was 1.85 years higher (95% CI: 0.67, 3.02). Inclusion of childhood social class as continuous confirmed a significant association of increasing Δage across groups (per-category Δage change = 0.39 year, 95% CI: 0.17, 0.61). For childhood social class, the addition of alcohol measures or psychological distress to the model reduced sample size and hence precision but did not affect conclusions (Table 3). For education, associations were partially explained by psychological distress (adjusted Δage = 0.78 year, 95% CI: 0.15, 1.70) and were fully explained by childhood social class (adjusted Δage = 0.56 year, 95% CI: −0.48, 1.60). In contrast, adjustment for education barely affected associations for childhood social class (Table 3).

Results for Horvath and Hannum age unadjusted for smoking and body mass index were very similar (Web Table 3). Additional adjustment for blood sample processing time (1 day, 2 days, or 3 days) did not affect results for either clock.

## DISCUSSION

In 1,099 men and women aged 28–98 years, we assessed associations of a range of socioeconomic position measures with DNAm age acceleration, to investigate the possible contribution of DNAm age acceleration to socioeconomic inequalities in health. We showed that Δage is primarily associated with socioeconomic position in childhood, rather than later in life. Documenting a negative relationship between Δage and chronological age, we showed that associations of Δage with the social environment may be vulnerable to substantial age-related confounding.

### Correlation of chronological and DNAm age across the adult life span

Chronological age correlated highly with DNAm age derived using both the Horvath and Hannum estimators. However, we found Δage to be robustly and negatively correlated with chronological age. Using the Horvath and Hannum estimators, older persons were almost exclusively “young” for their age, and using the Horvath clock, younger persons were almost exclusively “old” for their age. Precisely because DNAm age predicts mortality, the typically low Δage of older persons may result partly from survival bias. However, it is unclear how survival bias can explain the unexpectedly positive Horvath Δage among younger participants. Since participants for whom methylation was profiled needed to meet a range of criteria, including consenting to genetic analysis and participating in 12 annual surveys, an influence of other forms of bias was possible. However, these patterns persisted after we applied inverse-probability weights for nonresponse. This suggests that relationships of chronological age and methylation at sites included in the clocks may differ between populations, which has important implications for research using the clocks to investigate exposures or outcomes which are age-patterned. Researchers do not always adjust for chronological age when Δage is the outcome (26), but we find this can produce spurious associations of Δage with factors which are age-correlated. Since unemployment is disproportionately experienced by people early in their working lives, striking Δage elevations occur with aggregated duration of unemployment: with the Horvath clock, 3.16 years (95% CI: 1.81, 4.52) for less than 12 months and 3.66 years (95% CI: 1.68, 5.64) for 12 or more months; with the Hannum clock, 3.30 years (95% CI: 2.11, 4.50) for less than 12 months and 2.82 years (95% CI: 1.42, 4.21) for 12 or more months. These elevations disappear completely after adjustment for chronological age.

### Association of DNAm age acceleration with socioeconomic factors

In general, we observed accelerated epigenetic aging in relation to social disadvantage in childhood. Compared with participants with professional/managerial parents, a clear pattern was seen of increasing Hannum Δage for less advantaged groups; we also saw elevations in Horvath Δage for some less advantaged groups. Since smoking and adiposity may be mediators or confounders of these associations, results from health-behavior–adjusted models may be regarded as conservative. In any case, these differences were not explained by smoking, adiposity, or alcohol consumption, suggesting that mechanisms independent of health behaviors are involved.

The fact that associations between Hannum Δage and low education were explained by childhood social class, but not vice versa, supports early life as a critical period for establishment of DNAm age trajectories. Since there is some evidence that methylation sites in the Hannum clock may be more subject to stress-related processes (27), clearer patterns with the Hannum clock may implicate stress as a key factor in the social differences observed or may reflect the use of a DNAm age measure designed for whole blood, the same tissue used in this analysis. The fact that psychological distress did not explain associations may indicate that processes are largely independent of perceived distress or may reflect limitations of subjective psychological well-being measures in the study of socioeconomic inequalities.

Associations with childhood social class accord with results from the 1958 British Birth Cohort study (28) and research suggesting that childhood may be critical for establishment of DNAm age trajectories (29) and other aspects of DNA methylation (30). Since we were unable to examine particular aspects of the childhood environment that may plausibly affect DNA methylation aging—such as diet, housing quality, or psychosocial stress—further research will be required to identify which factors in childhood are most relevant to DNA methylation aging. It is also possible that childhood disadvantage is acting as a proxy for in utero conditions. Notably, since participants who at age 14 years had parents in professional occupations were younger (mean age = 55.4 years) than those whose parents were in semiskilled/unskilled occupations (mean age = 63.3 years), or without an employed parent (mean age = 58.8 years), any residual confounding by age itself is likely to have led to underestimation of associations with childhood social class. Thus, our estimates may be regarded as conservative.

Although childhood social class predicted adult socioeconomic position (Web Table 2), we saw no association with either current equivalized income or equivalized income over a 12-year period. This contrasts with results from a study of African-American women (8) but accords with a study of older Italians (10). Since our income measures were based on detailed, annually reported information, discrepancies are unlikely to have resulted from the quality of the income data. To investigate the contribution of sample sex composition, we repeated the income analyses with a sex interaction term, but we found no evidence of female-specific associations. It is possible that greater economic hardship experienced by participants in American samples (8) played a role. Our sample contained only white participants, but associations may differ by ethnicity; they may also differ between countries with different welfare provisions. Results for education and Horvath Δage are consistent with 2 recent analyses that found no robust associations (6, 31). Results for education and Hannum Δage are consistent with previously reported associations (6, 12, 31) but suggest that they may be partly explained by education’s acting as a proxy for unmeasured conditions earlier in life.

### Strengths and limitations

This study had several considerable strengths: Based on a national study, it comprised a large sample with representation from almost the entire adult age range. We applied inverse-probability weights to models, thus minimizing the impact of nonresponse bias, and annually repeated data collection minimized the impact of recall error on summary measures of income and unemployment across 12 years. We were able to consider diverse dimensions of socioeconomic position, finding that associations with education were explained by childhood social class. However, we could not examine conditions in early childhood or in utero, where effects on DNAm age trajectories are plausibly stronger than at age 14 years. The fairly crude measure of mental health available may not have adequately captured the contribution of psychological processes to DNAm age acceleration, and the sample was restricted to whites, meaning that results may not be generalizable to other ethnic groups. As with previous studies, the prediction of mortality by DNAm age acceleration means that survival bias may have produced underestimates of the impact of social exposures in older age groups.

### Conclusion

In a large sample of British study participants aged 28–98 years, DNAm age measured by means of the Horvath and Hannum clocks was associated with childhood social class but not with measures of social position later in life, which is consistent with a lasting influence of early-life conditions on DNAm age trajectories. Across the adult age range, population mean values of DNAm age “acceleration” varied substantially with chronological age. Studies examining associations with exposures and outcomes which themselves are age-patterned should take this into account to avoid age-related confounding.

## Supplementary Material

Web Material

## Abbreviations

CI: confidence interval
DNAm age: DNA methylation age
