# Supplementary material for: Socioeconomic Position and DNA Methylation Age Acceleration Across the Life Course
Source: Am J Epidemiol. 2018 Jul 27;187(11):2346–54. doi: 10.1093/aje/kwy155 (PMC6211240; doi:10.1093/aje/kwy155)
Supplement: Web Material [file kwy155_hughes_web_material_final.pdf]

Socioeconomic Position and DNA Methylation Age Acceleration Across the Life Course

Amanda Hughes, Melissa Smart, Tyler Gorrie-Stone, Eilis Hannon, Jonathan Mill, Yanchun Bao, Joe Burrage, Leo Schalkwyk, and Meena Kumari

Contents

Page 2: Web Table 1. List of missing probes

Page 3: Web Appendix: Inverse-Probability Weighting

Page 4: Web Table 2. Association of childhood social class with adult socioeconomic position measures in 2011–2012

Page 5: Web Table 3. Association of socioeconomic factors with DNA methylation age acceleration, not adjusted for smoking and body mass index, UK Household Longitudinal Study participants, 2011–2012

Page 6: Reference

Web Table 1. List of missing probes (probes included in Horvath and Hannum algorithms not profiled with the Illumina Infinium HumanMethylationEPIC array)

Horvath Missing Probes		
Probe ID	Chromosome	Gene Annotation
cg02654291	9	C9orf64
cg02972551	2	KDM3A;KDM3A
cg09785172	4	WFS1;WFS1;WFS1;WFS1
cg09869858	12	P11
cg13682722	14	C14orf102;C14orf102
cg16494477	5	FGF18
cg17408647	7	C7orf44;C7orf44
cg19273182	2	PAPOLG;PAPOLG
cg19945840	1	SDF4;SDF4;B3GALT6
cg27319898	7	ZNF804B;ZNF804B
cg04431054	5	PRRC1
cg05590257	17	PLD6
cg06117855	3	CLEC3B;CLEC3B
cg19046959	1	COL8A2
cg19569684	5	MGC29506
cg24471894	9	KIAA0020
cg27016307	19	HRC
cg02654291	9	C9orf64

Hannum Missing Probes		
Probe ID	Chromosome	Gene Annotation
cg24079702	2	FHL2;FHL2;FHL2;FHL2
cg14361627	7	KLF14
cg07927379	7	C7orf13;RNF32
cg18473521	12	HOXC4;HOXC4
cg09651136	15	PKM2;PKM2;PKM2
cg21139312	17	MSI2;MSI2

Web Appendix: Inverse-Probability Weighting

Weights for these analyses took into account initial sampling probabilities, attrition of BHPS participants prior to 2011, and differential nonresponse at the nurse visit, blood sampling and genetic/epigenetic sampling stages.

Inverse-probability weights for epigenetic analyses were constructed by building on existing weights by Lynn and colleagues (1). The base was a BHPS-specific longitudinal weight which takes account of initial sampling probabilities and assigns a non-zero value to BHPS participants who took part in the 2011–2012 nurse assessment and were present at mainstage interviews from 2001 to 2011. An add-on weight was created using information on demographic and health characteristics to take account of extra selection into the epigenetic sample, namely presence at two more BHPS waves in 1999 and 2000, inclusion at the blood sampling stage, and inclusion in genetic analysis. This was then combined with the existing weight. For sensitivity analyses involving alcohol consumption, this was further combined with an existing weight assigning non-zero values to participants who completed the self-completion section of the questionnaire at wave 2. For sensitivity analyses involving psychological distress at UKHLS wave 3, this was further combined with an existing weight assigning non-zero values to participants who completed the wave 3 self-completion questionnaire.

Web Table 2. Association[a] of childhood social class[b] with adult socioeconomic position measures in 2011–2012

	Age-specific current income quartiles (range 1-4; 1 = most advantaged)[c]		Number of times in the lowest income quartile, 1999–2011[d]		Total months unemployed, 1999–2011[e]		Age- and sex-standardized education (range 0-1; 1 = least educated)[f]	
	Coeff	95% CI	Coeff	95% CI	Coeff	95% CI	Coeff	95% CI
Professional/managerial (ref)	0	Referent	0	Referent	0	Referent	0	Referent
Skilled nonmanual	[g]0.28	0.03, 0.52	-0.23	-1.08, 0.61	0.29	-1.58, 2.16	0.03	-0.02, 0.09
Skilled manual	[g]0.42	0.25, 0.59	[g]0.73	0.15, 1.32	0.78	-0.49, 2.05	[g]0.10	0.06, 0.14
Semiskilled/unskilled	[g]0.65	0.44, 0.85	[g]1.63	0.92, 2.34	[g]2.66	1.11, 4.21	[g]0.19	0.14, 0.23
No parent employed/both deceased	[g]0.66	0.32, 1.00	1.03	-0.20, 2.25	[g]4.33	1.75, 6.91	[g]0.15	0.07, 0.23

Abbreviations: CI = confidence interval.

[a] Associations are from linear regressions of the adult socioeconomic position variable on childhood social class, age, age^2 and sex. [b] Parental Registrar General's Social Classification when participant was aged 14.

[c] N = 1026.

[d] N = 874.

[e] N = 1025.

[f] N = 1024.

[g] Results significant at $P < 0.05$.

Web Table 3. Association[a] of Socioeconomic Factors with DNA Methylation Age Acceleration, not adjusted for smoking and body mass index, UK Household Longitudinal Study participants, 2011–2012 (*N* = 1,094)

Quartiles of equivalized net household income (N = 1093/1094)	Horvath[a]		Hannum[a]	
	Δage (years)	95% CI	Δage (years)	95% CI
Highest (ref)	0	Referent	0	Referent
3	-0.94	-1.89, 0.01	-0.33	-1.02, 0.35
2	0.44	-0.48, 1.35	0.16	-0.57, 0.90
Lowest	-0.68	-1.58, 0.23	-0.11	-0.92, 0.70

Current employment status (participants aged <65, N = 716/717)	Horvath		Hannum	
	Δage (years)	95% CI	Δage (years)	95% CI
Employed (ref)	0	Referent	0	Referent
Self-employed	0.78	-0.42, 1.99	0.05	-0.97, 1.07
Unemployed	-1.18	-3.05, 0.70	-1.02	-2.27, 0.22
Retired	-0.53	-1.77, 0.70	-0.29	-1.33, 0.75
Looking after home or family	0.87	-0.48, 2.23	0.49	-0.61, 1.59
Long-term sick or disabled	2.07[b]	0.66, 3.48	-0.23	-1.69, 1.24
Other	1.61	-0.25, 3.46	1.17	-1.35, 3.69

Years in the lowest age-specific income quartile, 1999–2011 (N = 932/933)	Horvath		Hannum	
	Δage (years)	95% CI	Δage (years)	95% CI
0 (ref)	0	Referent	0	Referent
1-2	0.40	-0.49, 1.30	0.74	-0.09, 1.57
3-6	-0.49	-1.35, 0.37	0.05	-0.62, 0.71
7+	-0.54	-1.36, 0.28	-0.13	-0.84, 0.57

Total unemployment, 1999–2011 (N = 1091/1092)	Horvath		Hannum	
	Δage (years)	95% CI	Δage (years)	95%, CI
None (ref)	0	Referent	0	Referent
<12 months	-0.68	-1.64, 0.28	-0.39	-1.14, 0.37
≥12 months	-0.29	-1.65, 1.08	-0.92[b]	-1.77, -0.06

Highest educational qualification[c] (N = 1088/1089)	Horvath		Hannum	
	Δage (years)	95% CI	Δage (years)	95% CI
Least vs. most educated	0.44	-0.77, 1.66	1.01[b]	0.05, 1.96

Childhood social class[d] (N = 1025/1026)	Horvath		Hannum	
	Δage (years)	95% CI	Δage (years)	95% CI
Professional/Managerial (ref)	0	Referent	0	Referent
Skilled nonmanual	1.38[b]	0.21, 2.54	0.25	-0.55, 1.06
Skilled manual	0.50	-0.27, 1.26	0.68[b]	0.10, 1.26
Semiskilled/unskilled	0.96	-0.06, 1.98	1.08[b]	0.21, 1.95
No parent employed/both deceased	2.36[b]	0.68, 4.05	1.84[b]	0.70, 2.98

Abbreviation: CI = confidence interval.

[a] Adjusted for chronological age, age^2, sex, blood cell count, and batch.

[b] Associations significant at $P < 0.05$.

[c] Standardized within categories of sex and 5-year age group. Range 0−1; higher scores indicate lower education.

[d] Parental Registrar General's Social Classification when participant was aged 14.

Reference

1. McFall S, Petersen J, Kaminska O, et al. Understanding Society The UK Household Longitudinal Study Waves 2 and 3 Nurse Health Assessment, 2010 – 2012 Guide to Nurse Health Assessment. Colchester, UK: Institute for Social and Economic Research, University of Essex, 2014. URL: https://www.understandingsociety.ac.uk/documentation/health-assessment/user-guide. Accessed: 01/12/2017.
